# Recovery from a Wound in the Stomach

**Published:** 1838

**Authors:** Charles B. New

**Affiliations:** Rodney, Miss.


January 1838.

---

### Art. IV.—Wound of the Stomach.

*Case of Recovery from a Wound in the Stomach.* Communicated by Charles B. New, M. D., of Rodney, Miss., through Prof. Harrison.

An Indian received a stab in Natchez, on the 24th December, 1837. Six days elapsed before I saw him, during which period he walked to Rodney, which is thirty miles from Natchez. On the 30th of December I visited him, and found, upon examination, a wound of four inches long, a little below and to the left of the scrobiculus cordis. Protruding from the wound, there presented a tumour, which, upon first view, I thought was a portion of the bowels; but upon further inspection, I discovered that it was most probably omentum. The external surface of this mass was very vascular, and in a state of suppuration. There was so strong a demonstration of sphacelus in the tumour, that I determined upon its removal by ligature. Accordingly the ligature was drawn pretty tight about the tumour, close to the abdomen. In a short time after the application of the ligature I returned, and found him very ill. Incessant vomiting, small, rapid pulse, cold and clammy skin, indicated the necessity of removing the ligature. The knife was then resorted to for the removal of the protruding mass; but upon cutting into it, I found that a portion of the stomach constituted a part of the tumour. I carefully separated the already dead parts of the tumour from that which was not in a state of gangrene. In doing so, I had to remove a portion of the stomach. The stomach was secured by a ligature, and confined within the lips of the external wound. The wound was then stitched, and dressed with adhesive plaster.

On the 31st, I found him prostrated,—with cold skin, feeble pulse, nausea, and constipated bowels. The external wound, and that portion of the stomach which was perceptible through the wound, were considerably inflamed, but of a healthy aspect. Ordered him a solution of Epsom salts, with spirits nit. dulc. and tinct. opii camph.

Jan. 1. Some fever to-day, with nausea; his bowels were opened by the solution which he took yesterday. The external wound is suppurating. The edges of the wounded stomach very red, with slight suppuration.

2d. Free suppuration from the wound—appearances of granulation upon the thickened edges of the wounded stomach.

On the 5th of January, the ligature which secured the stomach came away. There is a firm adhesion of the stomach to the peritoneum along the wound.

10th. The wound in the integuments nearly closed by granulations. He is recovering rapidly; appetite is good, and bowels regular. On the 15th of January, he rose from his bed, the wound being almost entirely cicatrized; his appetite, digestion, and other functions of nutritive, as well as animal life, in a normal state.

